# Physician Attitudes on Integration of Prehospital Patient Care Report into Hospital Electronic Health Record

**DOI:** 10.5811/westjem.41540

**Published:** 2025-09-02

**Authors:** Maren Smith, Caroline Given, Soheil Saadat, Kenneth Leung, Julia Afrasiabi, Robert Katzer

**Affiliations:** * University of California, Irvine School of Medicine, Irvine, California; † University of California, Irvine Medical Center, Department of Internal Medicine, Orange, California; ‡ University of California, Irvine Medical Center, Department of Emergency Medicine, Orange, California

## Abstract

**Introduction:**

Prehospital information is valuable but often under-used by physicians. In both the emergency and inpatient settings, information about a patient’s condition prior to their arrival is important to provide optimal care. Historically, prehospital responders’ electronic patient care reports (ePCR) have not been integrated with the hospital’s electronic health record (EHR). In this study, we aimed to assess physician attitudes towards the ePCR and patient care decisions before and after integration of prehospital ePCR and hospital EHR systems. We hypothesized that this would increase accessibility and use of prehospital ePCR in patient care decisions.

**Methods:**

In 2023, our local academic health center implemented software that made prehospital documentation available to hospital staff within 30 minutes of patient arrival to the emergency department (ED). Before this, we surveyed attendings, fellows, and residents from both the ED and internal medicine (IM) department on their attitudes and behaviors regarding ePCR and clinical practice. We administered the same survey six months after implementation, and compared responses with a Wilcoxon signed-rank test.

**Results:**

Sixty-six physicians responded to the pre survey, including 39 (59.1%) from the ED and 27 (40.9%) from the IM department. Fifty-two completed the post survey, including 33 (63.5%) emergency physicians and 19 (36.5%) IM physicians. The pre- survey response rates were 92.9% and 54% for the ED and IM groups, respectively, while the post-survey response rates were 84.6% and 70.4%. Change in rank was significant (P < .01) for the following categories: knowledge;, ability; ease of use; time to access; and frequency of accessing the ePCR. Change in rank was not significant for the importance of ePCR in patient care and medical decision-making, and whether the ePCR would be used more frequently if it were easier to access.

**Conclusion:**

Pre- and post-survey responses regarding accessibility showed a significant change in rank, while the importance of the ePCR on clinical decision-making did not. This suggests that while system integration increased accessibility to prehospital information, it did not significantly alter patient care decision-making.

## INTRODUCTION

Structural challenges to efficient handovers between prehospital emergency medical service (EMS) responders and physicians in the emergency department (ED) are well-documented in the literature. Multifactorial barriers, including time limitations, high-acuity environment, and participation of multidisciplinary healthcare workers, have been noted in prior research.^1^–^3^ These challenges existed prior to the adoption of hospital electronic health records (EHR) and prehospital electronic patient care reports (ePCR). In fact, studies have demonstrated these challenges regardless of whether handover between EMS responders and physicians consists of verbal, electronic, or hardcopy communication tools.^2^, ^4^, ^5^

In 2015, legislation was passed in California requiring 9-1-1 prehospital responders to chart electronically. The ePCR allows EMS professionals to document demographics, vitals, assessment data, and any interventions performed en route to the hospital. This information ultimately generates a patient record that is digitally accessible to the receiving hospital.^6^ However, multiple studies have shown how this process is disrupted at the point of patient transfer, specifically when the ePCR must be downloaded, printed, and uploaded into the hospital EHR during patient registration.^4^, ^7^–^9^ This process obstructs efficient access by clinicians to the ePCR and encourages reliance on verbal handover or hardcopy printouts, leading to inconsistent, delayed, or omitted information sharing.^4^, ^5^, ^10^

In June 2023, our local academic health center adopted a new software that directly integrated the prehospital ePCR into the receiving hospital’s EHR, consequently forgoing this time-consuming clerical step. Although previous studies evaluating clinician attitudes toward the ePCR suggest that they would be more likely to use ePCR information if it were readily available,^7^, ^11^ there is little evidence to support this conclusion. Evidence is also sparse on clinician attitudes, their use of ePCR, and ePCR influence on patient care decision-making in response to ePCR/EHR integration. We sought to assess clinician attitudes toward ePCR accessibility and its role in patient care decision-making before and after ePCR/EHR integration. We hypothesized that this integration would increase accessibility and thus use of ePCR in patient care decision-making by in-hospital physicians.

## METHODS

### Study Setting and Population

We conducted a prospective, cohort, web-based survey of emergency physicians and IM physicians regarding use and accessibility of prehospital records; we collected and managed study data using Research Electronic Data Capture (REDCap) tools hosted at the University of California Irvine. This survey was administered before and six months after the ePCR/EHR integration. We established a protocol and developed a 15-question survey in which responses were ordered on a five-point Likert scale. Questions 1–2, 4–8, 10, and 13 gauged ePCR accessibility. The third question assessed how often clinicians received a direct verbal handoff from prehospital responders. Questions 9, 11, and 12 surveyed importance of ePCR to patient care. Questions 14 and 15 asked participants to identify their clinical role (resident, fellow, or attending) and department (ED or IM). Our institutional review board reviewed the protocol and granted self-exemption.

In June 2023, software that integrated prehospital ePCR and the hospital EHR went live. In May 2023, an invitation to complete the pre-software integration survey was sent to the emergency and internal medicine departments to assess pre-integration attitudes on ePCR accessibility and utilization. A period of six months was arbitrarily chosen to conduct the post survey. We considered this to be ample time for physicians to alter, if at all, their workflow. Participation was voluntary, and all participants were employed as residents, fellows, or attending physicians in the ED or IM department. Survey reminders were automatically sent out to those who did not complete the survey weekly for three weeks. To incentivize participation, all who completed the survey were automatically entered into a raffle to win one of five $20 gift cards.

**Population Health Research Capsule**

What do we already know about this issue?

*Prior studies suggest clinicians would be more likely to use information from prehospital electronic patient care reports (ePCR) if it were available*.

What was the research question?

*Does integration of prehospital patient charts with in-hospital health records affect clinician decision-making?*

What was the major finding of the study?

*There was no significant change in rank regarding importance of ePCR accessibility to patient care delivery*.

How does this improve population health?

*Our findings indicate that emergency medical services-to-emerency department information transfer is ineffective and suggests that electronic integration is a potential topic of research and solution*.

Six months later, in November 2023, an invitation to complete the post-software integration survey was sent to all who had completed the pre-integration survey. Similarly to the pre-survey, reminders were automatically sent weekly for three weeks to those who had not completed the survey. Additionally, all who completed the survey were automatically entered into a raffle to win one of five $20 gift cards.

### Statistical Analysis

Data are presented as count (percentage). By using a paired analysis approach, we compared respondents’ answers in the post-intervention survey to their pre-intervention survey answers. The changes are reported as negative ranks, positive ranks, and ties. The Wilcoxon signed-rank test was used to examine whether the change was statistically significant. Type I error was set to 5%. Following data collection, we performed data analysis using SPSS Statistics for Windows v28.0 (IBM Corp, Armonk, NY). We compared pre- and post-survey responses with a Wilcoxon signed-ranked test.

## RESULTS

### Pre-intervention Survey

**Figure 1:**
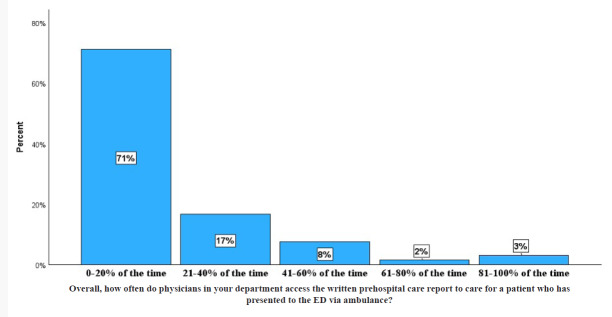
Responses of clinicians regarding their access to patient care summaries written by first responders prior to integration of prehospital and receiving hospital health records systems. *ED*, emergency department.

Sixty-six respondents, consisting of 35 (53.0%) residents, four (6.1%) fellows, and 27 (40.9%) attendings from the ED and IM department completed the pre-intervention survey (Table 1). The pre-survey response rates were 92.9% and 54%, respectively. Fifty-eight (87.9%) agreed or strongly agreed that having access to the ePCR is important for patient care in the ED or after admission. At the same time, 47 (71.2%) reported that physicians in their department have access to the written ePCR only 0–20% of the time (Figure 1).

Twenty-six (39.4%) respondents reported that 80% of the time physicians do not receive direct verbal report from an emergency medical technician (EMT) or paramedic. At the same time, 47 (71%) reported that if the physician did not receive a direct verbal report from the EMT or paramedic, they would only access the written ePCR 20% of the time; 47 (71.2%) were unaware that physicians could access the written ePCR; and 23 (34.8%) believed that accessing the written ePCR would take > 30 minutes. On the other hand, 62 respondents (93.9%) agreed that physicians would read the written ePCR more frequently, if it were easier to access.

### Post-intervention Survey

A total of 52 respondents returned the post-intervention survey (Table 1). The post-survey response rates were 84.6% and 70.4% for EM and IM groups, respectively. Nine had a change in their clinical role compared to the pre-intervention period.

Table 2 shows the statistically significant change in respondents’ answers from the pre- to post-intervention period. We did not observe a statistically significant change in respondents’ answers from the pre- to post-intervention period with regard to the following survey items: 1) How often are physicians able to access the written ePCR and/or receive verbal report from the EMT or paramedic before they examine the patient? (P = .05); 2) Is having access to the written ePCR important for patient care in the ED and/or during hospitalization (P = .53); and 3) Would physicians read the written ePCR more frequently if it was easier to access (P = .59); 4) Over the past three months, how often did you make important patient treatment decisions based on verbal report from EMT or paramedic (P = .12); and 5) Over the past three months, how often did you make important patient treatment decisions based on the written ePCR (P = .24).

When stratifying the results based on department, there was no significant change in ranking regarding attitudes toward the importance of ePCR accessibility to patient treatment decisions in either the EM or IM groups. Similarly, when stratifying the results based on role, there was no significant change in rank regarding importance of ePCR accessibility to patient care delivery in either the resident or attending groups.

## DISCUSSION

This study looked at whether implementing an electronic interface between prehospital ePCR and the receiving hospital EHR affected physician perspectives on access to the ePCR. Additionally, we assessed perceived differences in patient treatment decisions in response to ePCR/EHR integration. This integration resulted in both a more consistent understanding of how to access the ePCR as well as increased use of the ePCR.

Previously, research identified that valuable information is lost during the EMS to ED handoff process.^3^ In systems that transitioned from handwritten PCRs to ePCRS, paramedics indicated that they often still feel the need to provide additional information at the time of verbal handoff. 11 Furthermore, prior studies have demonstrated that physicians prefer ePCRs over handwritten PCRs for reasons of legibility and accuracy. Of note, the printed version of that ePCR was usually not available at the time of physician patient assessment.^7^,^10^ One study identified the processes that contributed to a system’s failure to make the printed version of the ePCR available to physicians. Challenges included coordination of both the external processes of paramedics completing the ePCR, the ePCR being faxed to the ED, and the manual internal process of the unit clerk retrieving the faxed report and placing it in the patient record.^8^ A study evaluating the challenges to effective EMS to ED patient care handoffs identified harnessing technology as one way to close the gaps in this information exchange.^5^ We believe that the direct automatic integration of the ePCR into the receiving hospital’s EHR uses technology to address many of the identified shortcomings of the current EMS to ED patient handoff.

Our pre-software integration surveys demonstrate that physicians at our institution experienced similar challenges with accessing the written ePCR to those previously documented in the literature. Additionally, our results suggest that our software interface succeeded in improving reported physician knowledge of how to access the ePCR as well as reported physician ease of access to the ePCR as demonstrated by the significantly decreased reported time in which physicians can access the ePCR. These findings are significant as they address many of the barriers to efficient EMS to ED handoff previously reported in the literature.

Our study does not demonstrate that increased access to the written ePCR resulted in increased perception that prehospital information influenced important patient care decisions. This finding will require further inquiry. An important function of the ePCR should be to provide the physician with the information needed to make important patient care decisions. This study does not answer the question of why physicians did not have that perception. Some possibilities include that physicians historically have not used the printed PCR and have yet to fully integrate the electronic version into their decision-making process. It may be that the physicians do not trust the assessments of EMTs and paramedics to the extent that they use them for important patient care decisions. Furthermore, it may be that the information on the ePCR corresponds to the physician’s assessment of the patient and, therefore, does not change the management decisions. Regardless, further study is required.

## LIMITATIONS

Our study had several limitations. As a convenience sample of emergency physicians and internal medicine physicians at our institution, the risk exists for selection basis of those who participated as compared to those who chose not to participate. Furthermore, 14 fewer people responded to the post- than the pre- intervention survey, and this may have resulted in selection bias of the post-intervention group. We worked to minimize these effects by sending several reminder emails to encourage physicians to participate. A second potential limitation is that the physicians’ perceptions of the system and its use may not reflect actual practice. To mitigate this, our methods included many questions phrased to elucidate perceptions of the group and not necessarily individual perceptions. Finally, the culture of high-quality EMS handoffs in this ED specifically should be noted as a potential confounding factor as to why physicians viewed ePCRs to be of low importance regarding patient care decision-making.

## CONCLUSION

Overall, pre- and post-survey responses regarding accessibility to prehospital patient data did exhibit a significant change in rank while the importance of the electronic patient care report in clinical decision-making did not differ significantly. This suggests that while integrating prehospital ePCR and hospital electronic health record systems increased accessibility to prehospital information, this increase in access did not significantly alter patient care decision-making by in-hospital physicians. These findings align with the consensus in the literature on the ineffectiveness of this information transfer and, more importantly, suggest electronic integration as a potential solution to this problem. However, more research is needed to better understand physician use patterns of prehospital information so that it may be optimized. The consistent ease of access to ePCRs will hopefully foster a better understanding of its utility for clinicians and likely present opportunities for future focused studies.

## Figures and Tables

**Table 1:** Clinical roles of participants in pre- and post-intervention surveys regarding use of prehospital patient data.

		Pre-/Post-intervention survey			
	
		Pre-intervention		Post-intervention	
	
		Count	Percentage	Count	Percentage
	
Respondents’ role*	Resident	35	53.0%	21	40.4%
	Fellow	4	6.1%	6*	11.5%
	Attending	27	40.9%	25*	48.1%
Department	Emergency medicine	39	59.1%	33	63.5%
	Internal medicine	27	40.9%	19	36.5%
		66	100.0%	52	100.0%

* Nine respondents in the post-intervention survey had changed their clinical roles since completing the pre-intervention survey.

**Table 2:** The change in respondents’ answers from the pre- to post-intervention period regarding access to first responders’ patient care reports.

		N	Sum of Ranks	P-value
Physicians know how to access the written ePCR for a patient presented ED via ambulance.	Negative Ranks	0	0.00	< .001
	Positive Ranks	36	666.00	
	Ties	16		
Physicians can access a copy of the written ePCR.	Negative Ranks	1	5.00	< .001
	Positive Ranks	27	401.00	
	Ties	24		
How often do physicians receive direct verbal report from the EMT bringing the patient?	Negative Ranks	3	31.00	.02
	Positive Ranks	14	122.00	
	Ties	35		
If physicians do not receive direct verbal report from the EMT bringing the patient, it is easy for them to access written ePCR.	Negative Ranks	2	47.50	< .001
	Positive Ranks	37	732.50	
	Ties	13		
If physicians do not receive a direct verbal report from the EMT bringing the patient, how often do they access the written ePCR?	Negative Ranks	6	118.00	< .001
	Positive Ranks	31	585.00	
	Ties	15		
How long does it take physicians access the written ePCR for a patient brought via ambulance?	Negative Ranks	32	719.00	< .001
	Positive Ranks	9	142.00	
	Ties	11		
How often do physicians access the written ePCR to care for a patient who is brought via ambulance?	Negative Ranks	8	119.00	.01
	Positive Ranks	24	409.00	
	Ties	20		
If you did not receive direct verbal report from the EMT, it is easy to determine whether the patient presented to the ED via ambulance.	Negative Ranks	7	81.00	< .01
	Positive Ranks	21	325.00	
	Ties	24		

* Only statistically significant changes in rank are included.

*ePCR*, electronic patient care report; *ED*, emergency department; *EMT*, emergency medical technician.
